# Attendance, Weight Loss, and Participation in a Behavioural Diabetes Prevention Programme

**DOI:** 10.1007/s12529-022-10146-x

**Published:** 2023-01-11

**Authors:** Stavros Poupakis, Maria Kolotourou, Harry J. MacMillan, Paul M. Chadwick

**Affiliations:** 1 Institute for Global Health, University College London, 30 Guilford Street, London, WC1N 1EH UK; 2 Discover Momenta, Woodstock, UK; 3 Centre for Behaviour Change, University College London, London, UK

**Keywords:** Diabetes prevention programme, Weight loss, Behavioural change, Health inequalities

## Abstract

**Background:**

Weight loss in diabetes prevention programmes has been shown to be associated with participants’ age, socio-economic status, and ethnicity. However, little is known about how these differences relate to attendance and whether such differences can be mediated by other potentially modifiable factors. Differential effectiveness across these factors may exacerbate health inequalities.

**Method:**

Prospective analysis of participant data collected by one provider of the standardised national NHS diabetes prevention programme in England. Mediation analysis was performed via a structural equation model to examine whether the number of attended sessions mediated the associations of age, socio-economic status, and ethnicity with follow-up weight. The group-level factor of number of attended sessions was examined using multiple linear regression as a benchmark; multilevel linear regression using three levels (venue, coach, and group); and fixed effects regression to account for venue-specific and coach-specific characteristics.

**Results:**

The associations of age, socio-economic status, and ethnicity with follow-up weight were all mediated by the number of attended sessions. Group size was associated with attendance in an inverted ‘U’ shape, and the number of days between referral and group start was negatively associated with attendance. Time of day, day of the week, and the number of past groups led by the coach were not associated with attendance.

**Conclusion:**

Most of the differences in weight loss initially attributed to socio-demographic factors are mediated by the attendance of the diabetes prevention programme. Therefore, targeted efforts to improve uptake and adherence to such programmes may help alleviate inequalities.

## Introduction

The increasing rate of Type 2 diabetes is a public health concern, putting pressure on health systems globally [1]. Lifestyle programmes aiming to support behavioural change to prevent the onset of Type 2 diabetes have been an effective way to reduce this incidence [2, 3]. Pragmatic diabetes prevention interventions can achieve this, but their effectiveness depends on the degree to which participants adhere to programme guidelines [4, 5]. In England, the prevalence of Type 2 diabetes has been on the rise, with its rate in 2020 being at 4.7% [6]. In 2016, the National Health Service (NHS) established a universal Diabetes Prevention Programme (NHS-DPP) targeting people who have nondiabetic hyperglycaemia. The delivery of the NHS-DPP is conducted by independent providers that deliver the service to according to specific guidelines set by the NHS [7].

Research into the fidelity of design of the NHS-DPP shows that the programme is largely delivered according to the guidelines set out by the Department of Health and Social Care [8, 9]. However, programme delivery of the NHS-DPP varies substantially and this variation has implications for the patient experience, especially in terms of venue quality, scheduling, and group size, which have been proven important factors of participant satisfaction with programme [10]. Substantial variation is also observed in staff training [11]. All these delivery characteristics affect patient experience and therefore potentially affect adherence to the intervention.

Whilst there are established differences in the effectiveness of the NHS-DPP across age, socio-economic status, and ethnicity [12], little is known about what causes such differential responses. There are several possible modifiable factors that may contribute to this, including aspects of the intervention itself such as the size of the group or skills of the person delivering the intervention (implementation-level factors), as well as aspects of the way the individuals respond to the intervention (e.g. degree of attendance and adherence to behavioural recommendations). Implementation and participant behavioural factors may be potentially modifiable and therefore represent an opportunity to reduce or eliminate responses to the NHS-DPP that contribute to inequalities [13].

Adherence to behavioural interventions is an important component of their effectiveness. In the NHS-DPP there are several ways in which participants can be said to adhere to the intervention including attendance and the degree to which they implement the multiple behavioural recommendations for lifestyle change. Although adherence to behavioural recommendations is likely to be most strongly related to outcome, this is difficult to measure. As recommendations may vary across different providers of the NHS-DPP, benchmarking across providers is challenging. Session attendance is a critical measure of participant adherence and previous research has demonstrated it has a significant impact on the effectiveness of behavioural interventions, such as diabetes prevention or weight management programmes [12, 14–16]. Higher attendance has been strongly associated with greater weight loss in several trials [17–19]. Session attendance can be measured across programmes and therefore it may be a good candidate for benchmarking performance relating to inequality across programmes. Whilst differences in participation across sociodemographic groups in behavioural interventions are well established, little research has explored whether group-level modifiable factors, such as group size, time and place, are associated with differential participation using prospective models [20].

The way NHS-DPPs are implemented may also be related to differential outcomes between groups. Process evaluations of the DPP suggest several candidates for factors that might influence participant experience, engagement, and adherence to the NHS-DPP [21]. The delivery of these behavioural interventions takes place within groups, usually referred by primary care practises. Thus, in addition to individual-level characteristics, there are aggregate factors that affect the uptake and participation to these programmes, such as referring practise characteristics [22] or characteristics of the way the programme is delivered by coaches [10].

This study aims to identify potentially modifiable factors influencing the differential socio-demographic response to the NHS-DPP. Using data from one provider of the NHS-DPP, this study examines whether the number of attended sessions mediates the associations of individual-level characteristics with weight loss. In addition, some group-level factors of the number of attended sessions are examined to provide guidance for future programme design.

## Methods

The data used in this study were provided by one of the four commercial providers of the framework 1 of the NHS-DPP, Reed Momenta. This involved extracting anonymised data on the eligible individuals that were referred to participate to the NHS-DPP through this provider. These referred individuals were invited for an initial assessment and then booked into a group led by a coach at a designated venue to follow the 18-session programme over 9 months.

This study design provides different sources of variation in data collection that we exploit to explore group-level factors of attendance. Specifically, each venue hosted multiple groups, some of which started at the same date. Coaches were assigned to multiple groups, some within the same venue, and others at different venues. Whilst the participant allocation to the venue is mostly driven by geographical criteria and is highly endogenous, the participant allocation to the group within the venue is largely based on scheduling and resources relating the venue and the coach.

The outcome variables examined are follow-up weight in kgs (weight at last session attended) and the number of sessions attended. Predictors include participant-level and group-level characteristics. The participant-level predictors used are sex, age in 5-year groups, Index of Multiple Deprivation (IMD) quintile (an area-level measure of relative deprivation calculated for each of the Lower Layer Super Output Areas in England across the following domains: income; employment; health deprivation and disability; education, skills training; crime; barriers to housing and services; and living environment), and self-reported ethnicity. As we are interested in weight change, we also include weight at the first session in the regression. With the inclusion of initial weight, the analysis of follow-up weight is effectively examining weight change. The group-level variables are the following: group size and group size squared (to test for non-linearities) based on the number of participants that started each group; groups per coach based on the number of groups that each coach has delivered up until the current group, starting with the value of one and increasing by one with each subsequent group; number of days between referral and group starting date (in logarithm as this is highly skewed and has no zero values), time of the day, day of the week sessions run, and dummy variables for region and year (to account for regional differences and time differences, respectively).

The analysis in this study is divided in two parts. The first part explores these individual-level factors using a mediation analysis to examine whether the number of attended sessions mediated the associations of the participant-level characteristics with follow-up weight. The second part explores these group-level factors, by examining their relationship with attendance whilst controlling for the individual-level factors and accounting for unobserved heterogeneity at various levels in the implementation.

**Fig. 1:**
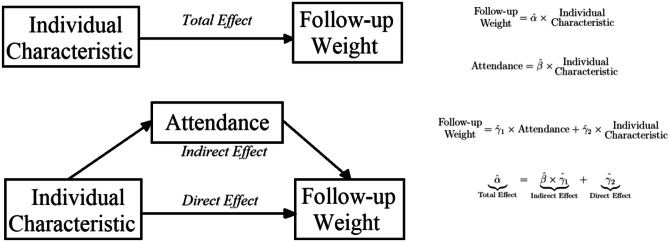
Mediation analysis graph and equations for estimated models

The mediation analysis is performed on follow-up weight (outcome), sessions (potential mediator), and the individual-level characteristics, calculating total, direct, and indirect effects of the individual-level characteristics. This was done using the Baron and Kenny approach [23], where the shares of the effects that are mediated by sessions can be calculated as the ratio of indirect over total. Figure 1 provides a graph of the mediation analysis strategy along with equations of the models that are estimated to retrieve all three effects. Inference was performed via bootstrap replications, as is standard in mediation analysis [24].

For the analysis of the group-level factors, the current design allows us to account for unobserved heterogeneity at various levels, by accounting for unobserved venue- and coach-specific characteristics. For example, groups in a small church in the city centre might be smaller in size compared to groups in a large modern community centre in a suburban area. By exploiting the fact that we have multiple groups within the same venue, we account for venue-specific characteristics (which may relate to the large geographic and socio-economic disparities) and estimate within-venue effects. Similarly, we account for coach-specific characteristics (sex, age, education, motivation, etc.) and estimate the effect of coach delivery experience (proxied by the number of groups the coach had started before the group’s first day). We present three specifications, using linear regression analysis as a benchmark, multilevel (mixed-effects) linear regression, and a fixed effects estimation (within estimator). All statistical analysis was conducted in Stata [25].

## Results

**Fig. 2:**
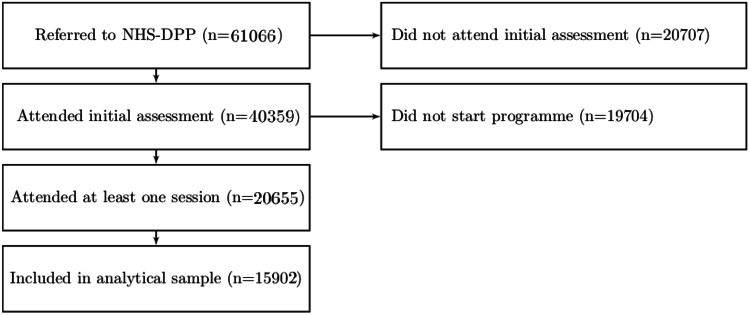
Flowchart of participants

Between June 2016 and December 2019, 61,066 eligible individuals were referred to Reed-Momenta to participate in the NHS-DPP. Of those, 40,359 (66%) attended an initial assessment and 20,655 (34%) attended at least one session (i.e. started the programme). Those with missing information on weight were excluded, resulting to an analytical sample of 15,902 individuals. These were referred from 1314 General Practitioner (GP) practises, with 2055 groups delivered in 330 venues (mainly in London, North-West, and South-East) from a total of 147 coaches. The flowchart in Fig. 2 shows the number of participants that remain or dropout at each stage of the recruitment process and those included in the analytical sample.

Each venue hosted an average of six groups. Conditional on starting on the same day, the average number of groups was two per venue, whereas conditioning on starting the same month, the average was 2.5 per venue. Each venue had an average of three coaches. Each coach instructed, on average, in about six venues and to 13 groups, with each group having an initial size of about 11 participants. Table 1 presents descriptive statistics for all variables used in the analysis.

**Table 1:** Summary statistics of the variables used in the analysis

	Mean (SD), *n* (%)
Follow-up weight	81.3 (18.4)
Weight at first session	83.7 (18.7)
Number of sessions attended	9.8 (5.8)
Sex	
Female	8734 (55%)
Male	7168 (45%)
IMD	
Q1—Most deprived	3020 (19%)
Q2	3046 (19%)
Q3	2777 (17%)
Q4	3494 (22%)
Q5—Least deprived	3565 (22%)
Age	
18–40	375 (2%)
40–45	395 (2%)
45–50	672 (4%)
50–55	1096 (7%)
55–60	1516 (10%)
60–65	2006 (13%)
65–70	2933 (18%)
70–75	3211 (20%)
75 +	3698 (23%)
Ethnicity	
White	10,315 (65%)
Asian	1107 (7%)
Black	806 (5%)
Other mixed	557 (4%)
Missing ethnicity	3117 (20%)
Group size	14.1 (6.3)
Groups per coach	9.3 (9.7)
Log (Days referral to start)	4.4 (0.8)
Day of the week	
Monday	3432 (22%)
Tuesday	3459 (22%)
Wednesday	2929 (18%)
Thursday	2791 (18%)
Friday	2443 (15%)
Saturday	848 (5%)
Time of the day	
9.00 or 9.30	1506 (9%)
10.00 or 10.30	2784 (18%)
11.00 or 11.30	2483 (16%)
12.00 or 12.30	1794 (11%)
13.00 or 13.30	2214 (14%)
14.00 or 14.30	1672 (11%)
15.00 or 15.30	1112 (7%)
16.00 or 16.30	931 (6%)
17.00 or 17.30	570 (4%)
18.00 or 18.30	836 (5%)

Calculations based on analytical sample (*n* = 15,902)

The results of the mediation analysis of follow-up weight and sessions attended are presented in Table 2. In the total-effects analysis, there are strong significant associations of follow-up weight with age, IMD quintile, and ethnicity, but not with sex. Participants of older age, higher IMD, Asian, other mixed, and missing ethnicity, have a greater weight loss. All these predictors are associated with more sessions attended, except for ethnicity. In the direct-effects model which includes the individual predictors and sessions, none of these predictors remains significant and their magnitudes are close to zero. Moreover, every session attended is associated with a weight loss of 0.281 kg (*p* < 0.001). Initial weight remains a strong predictor in both total- and direct-effects models.

**Table 2:** Mediation analysis for the relationship between follow-up weight and age, socio-economic status, and ethnicity, using the attendance as a mediator

	Sessions attended	Weight			
	Mediator	Total	Direct	Indirect	% Mediated
	Coeff. (S.E.)	Coeff. (S.E.)	Coeff. (S.E.)	Coeff. (S.E.)	
Number attended sessions			− 0.281***		
			(0.006)		
Weight at first session	− 0.010***	0.953***	0.950***		
	(0.003)	(0.003)	(0.003)		
Male	0.059	0.042	0.059		
	(0.097)	(0.079)	(0.075)		
Age 40–45	0.824**	0.042	0.273		
	(0.388)	(0.275)	(0.261)		
Age 45–50	0.703**	− 0.145	0.052		
	(0.346)	(0.265)	(0.250)		
Age 50–55	1.533***	− 0.474**	− 0.044	− 0.430	91%
	(0.325)	(0.242)	(0.226)	(0.092)	
Age 55–60	1.814***	− 0.809***	− 0.300	− 0.509	63%
	(0.315)	(0.232)	(0.218)	(0.089)	
Age 60–65	2.514***	− 1.073***	− 0.368*	− 0.705	66%
	(0.309)	(0.224)	(0.210)	(0.089)	
Age 65–70	3.226***	− 1.384***	− 0.479**	− 0.905	65%
	(0.304)	(0.218)	(0.205)	(0.087)	
Age 70–75	2.937***	− 1.268***	− 0.444**	− 0.824	65%
	(0.306)	(0.215)	(0.202)	(0.088)	
Age 75 +	2.313***	− 0.981***	− 0.332*	− 0.649	66%
	(0.306)	(0.213)	(0.199)	(0.087)	
IMD Q2	0.740***	− 0.262**	− 0.055	− 0.208	79%
	(0.150)	(0.117)	(0.109)	(0.042)	
IMD Q3	0.987***	− 0.357***	− 0.081	− 0.277	78%
	(0.159)	(0.130)	(0.122)	(0.045)	
IMD Q4	1.110***	− 0.399***	− 0.087	− 0.312	78%
	(0.153)	(0.123)	(0.114)	(0.043)	
IMD Q5—Least deprived	1.309***	− 0.584***	− 0.217*	− 0.367	63%
	(0.152)	(0.127)	(0.120)	(0.043)	
Asian	− 1.479***	0.518***	0.103	0.415	80%
	(0.198)	(0.134)	(0.128)	(0.056)	
Black	0.598***	0.181	0.349*		
	(0.223)	(0.195)	(0.188)		
Other mixed	− 1.036***	0.367*	0.077	0.291	79%
	(0.254)	(0.211)	(0.206)	(0.072)	
Ethnicity missing	− 0.678***	0.345***	0.154*	0.190	55%
	(0.121)	(0.095)	(0.089)	(0.034)	
Region and year dummies	Included	Included	Included		

Columns 1–3 report coefficients estimated via linear regression. Column 4 reports coefficients estimated using the Baron Kenny method. Column 5 shows % mediated calculated as indirect/total. Robust standard errors shown in parentheses. ***, **, and * indicate *p* < 0.01, *p* < 0.05, and *p* < 0.10, respectively. Reference categories for categorial variables: female (sex); Age 18–40 (age); IMD Q1—Most deprived (IMD); White (Ethnicity). Calculations based on analytical sample (*n* = 15,902)

**Fig. 3:**
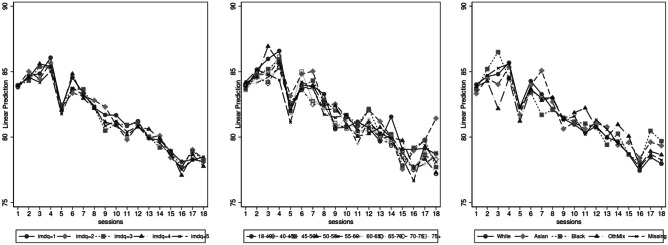
Weight loss predictions based on interactions of sessions attended with age, socio-economic status, and ethnicity

Indirect effects (and proportion mediated) are calculated for those categories which have a statistically significant total effect, as otherwise there is no effect in the first place (except for initial weight which is included only as a control). The large indirect effects reveal that for age, IMD, and ethnicity, their associations with follow-up weight are mediated via sessions attended, in most cases at about 70%. This is also confirmed in a fully interacted model of attended sessions and the three individual-level predictors (Fig. 3).

Examining the associations with group-level variables, all models indicate strong associations with group size and days between referral and programme start dates (Table 3). The benchmark model also shows a positive association with groups-per-coach, the 3 pm time slot, and Saturday. These associations are not present in the two models that account for unobserved heterogeneity. The positive sign of the linear term and negative sign of the quadratic term of group size reveal a relationship with the number of sessions attended that has an inverted ‘U’ shape, where the maximum is reached at between 15 and 18 participants. The logarithm of the number of days between referral and programme start has a strong negative relationship, suggesting that an increase in days by 100% (doubling the time between referral and start) is associated with a decrease of 0.3 sessions attended (*p* < 0.001). The groups-per-coach has no relationship with sessions attended, and the same is true for time of the day and day of the week.

**Table 3:** Associations of number of sessions attended with potentially modifiable group-level factors

	Linear Regression	Multilevel regression	Fixed effects regression
	Coeff. (S.E.)	Coeff. (S.E.)	Coeff. (S.E.)
Group size	0.347***	0.228***	0.190***
	(0.030)	(0.044)	(0.035)
Group size squared	− 0.010***	− 0.007***	− 0.006***
	(0.001)	(0.001)	(0.001)
Groups per coach	0.013**	− 0.026	− 0.019
	(0.006)	(0.019)	(0.018)
Log(Days Referral to Start)	− 0.221***	− 0.276***	− 0.282***
	(0.061)	(0.061)	(0.062)
9 or 9.30 am	0.379	0.627	0.306
	(0.253)	(0.426)	(0.318)
10 or 10.30 am	0.119	0.402	0.253
	(0.234)	(0.393)	(0.297)
11 or 11.30 am	− 0.099	0.214	− 0.010
	(0.238)	(0.399)	(0.298)
12 or 12.30 pm	− 0.009	0.310	0.0734
	(0.248)	(0.411)	(0.303)
1 or 1.30 pm	− 0.231	− 0.089	− 0.094
	(0.241)	(0.401)	(0.303)
2 or 2.30 pm	− 0.174	0.002	0.087
	(0.250)	(0.412)	(0.305)
3 or 3.30 pm	− 0.701***	− 0.505	− 0.502
	(0.269)	(0.438)	(0.327)
4 or 4.30 pm	− 0.408	− 0.328	− 0.447
	(0.276)	(0.453)	(0.335)
5 or 5.30 pm	0.124	0.320	0.229
	(0.317)	(0.481)	(0.348)
Tuesday	0.0357	− 0.0347	0.050
	(0.140)	(0.232)	(0.199)
Wednesday	0.0839	− 0.0594	− 0.007
	(0.148)	(0.242)	(0.206)
Thursday	− 0.267*	− 0.301	− 0.180
	(0.149)	(0.246)	(0.220)
Friday	0.099	0.051	0.201
	(0.153)	(0.266)	(0.251)
Saturday	0.415*	0.621	0.384
	(0.235)	(0.432)	(0.406)
Controls	Included	Included	Included

Columns 1–3 report coefficients estimated via the respective regression mentioned in the first row. Robust standard errors shown in parentheses. ***, **, and * indicate *p* < 0.01, *p* < 0.05, and *p* < 0.10, respectively. Controls include initial weight, sex, age, IMD quintile, ethnicity, contract region, and year dummies. Reference categories for categorial variables: 6 or 6.30 pm (Time of the day); Monday (Day of the week). Calculations based on analytical sample (*n* = 15,902)

## Discussion

This study aimed to identify potentially modifiable characteristics of the implementation or participant response to an NHS-DPP relating to differential effects between social and demographic groups. Greater attendance to the programme was associated with a lower follow-up weight during the programme, indicating a dose–response relationship. A decrease in follow-up weight of 0.28 kg per session attended was found in the current study. This is similar to a larger evaluation of the NHS-DPP that found a 0.32 kg weight loss per session [12], and other commercial weight management programmes, with the attendance-weight loss relationship holding for both for referred and self-referred programmes [15, 16, 19]. Whilst this relationship is evident, the mechanism via which this happens remains unclear. Attendance might be related to programme adherence or other behavioural components, which may differ between people [16]. Indeed, a systematic review of weight loss interventions failed to reach conclusion towards a consistent set of factors that predict dropout [26]. This may be because such psychological and behavioural determinants are usually largely unobserved and confound the analysis.

Age was an important predictor of attendance, with older participants attending far more sessions. Other studies have shown a similar pattern of attendance with age [19, 22, 27, 28]. The relationship with ethnicity and deprivation is more mixed in the literature, with some studies finding evidence of differences, whilst others do not [13, 19, 29], but this may depend on the type of measurement, especially in terms of measuring deprivation. A study that also used the IMD in Scotland found a similar pattern of decreasing attendance with increasing deprivation [22]. In line with other studies [19, 22], there was no difference in attendance between men and women.

The present finding of an association between individual characteristics and the number of sessions attended are in line with the results of a study which sampled data from all NHS-DPP providers that looked at predictors of initial attendance and of completion [12]. Lower attendance rates were found for younger participants, those from the most deprived quintiles, and for Asian and mixed ethnic groups, but not for sex.

This study found that attendance was associated with group size following an inverted ‘U’ shape, with maximum attendance for participants in groups with around 15–18 people. This is consistent with previous work showing group size is an important determinant of participant’s experience. In a qualitative evaluation of the NHS-DPP, participants in groups of 10–15 people reported more positive responses, compared to participants in groups of more than 15 people [11]. Another study on paediatric weight management interventions found that engagement was lower for participants in groups with more than 20 members, compared to groups with less than 20 members [30]. The results of the present study provide empirical support to suggest the maximum group size for optimising attendance and intervention effectiveness should be between the recommendations of 15 people by the National Institute for Clinical Excellence (NICE) [31] and 20 people by the NHS [7]. It is not known why larger group sizes may contribute to inequalities. One possibility is that coaches in larger groups may not have enough time to identify and deal with the specific needs of individuals from lower socioeconomic status and non-white ethnicities, resulting in lower engagement and premature dropout. Further research is needed on this issue.

Heterogeneity in coaches is another potential source of variation in adherence among groups. There are several sources of heterogeneity between coaches, such as professional status, delivery style, and experience. In this study, we used coach experience as one potential source of heterogeneity as previous systematic reviews on the effectiveness of the diabetes prevention programme in the USA, showed no differences when intervention delivered by clinically trained professionals or lay educators [32], and data on delivery style was not available. In the current study, the positive association in the benchmark model did not remain in neither of the main models. That is that although there are differences between coaches with high number of groups and coaches with low number of groups, within-coach, in other words, accounting for unobserved heterogeneity in coaches’ characteristics, groups-per-coach had no association with attendance. This result can be translated that there is no learning curve (nor fatigue) during the duration of the programme. The lack of information on coaches’ backgrounds, including prior experience between this programme, limits the interpretations of the analysis into just that. It is worth noting that, at the time, coaches had only limited mentoring, quality assurance and management support.

The timing of the sessions is also an important component in the delivery of such programmes. Indeed, research which aggregated data across four NHS-DPP providers showed that the providers with the lowest completion rates were the ones with more scheduling issues [10]. Results from the benchmark model suggest a possible decrease in attendance at 3 pm, which coincides with schools’ closure time, and a possible increase in attendance on Saturdays, possibly as this is part of the weekend. Interestingly, these differences vanish in the main models. Overall, the null effects of time of the day and day of the week in this study can be interpreted in two ways. Either scheduling does not matter for attendance, or participants self-selected (or session organisers selected) into the most appropriate schedule, thus minimising the disturbance in the daily or weekly routine. The negative association of the number of days between referral and programme start is indicative of the importance of timing, as delays might negatively affect individuals’ motivation which is an important determinant of participation [33].

The present study is unique in its focus on understanding potentially modifiable factors influencing the differential effectiveness of the NHS-DPP. Using appropriate methods to account for unobserved heterogeneity due to venue- and coach-specific characteristics, a set of clear policy suggestions are made, that are easy to implement by providers of similar programmes. We did not implement multiple imputations in the analysis, as the independent variables have no missing data (except ethnicity for which a separate category was created and included). Thus, we do not expect the incomplete cases to have any beneficial contribution. Indeed, previous analysis of this data found no differences in the sign and magnitude between complete-case and multiple imputations [12]. However, this study has a number of limitations. First, it uses data only from one of the four programme providers, which was slightly different in that it offered 18 sessions, whilst the others offered 13 in total. Second, participants self-select themselves to the sessions they attend, so the dose–response relationship and the mediation analysis results may be driven by factors associated with this selection, such as own motivation. Third, attendance reflects only the total number of sessions attended. Additional information on the exact sessions that each participant attended could reveal patterns of attendance, especially since weight loss from session to session is an important factor of subsequent dropout [34]. Fourth, the lack of HbA1c information, as low values of HbA1c measurement during the programme is another potential predictor of dropout [35]. The provider aimed to measure HbA1c at substantially less frequent intervals than weight, thus ruling out any analysis of the association between HbA1c change and sessions. This can be problematic, as some individuals who have prediabetes might have weight in the healthy range, but still, a target of modest weight loss would still result in clinically significant outcomes (36). Fifth, other coach characteristics, such as prior experience may be important factors that affect attendance. Sixth, despite accounting for venue-specific characteristics, the lack of randomisation in group allocation, may still hinder estimation of the relationships between the group-level characteristics examined in this study.

This study examined the relationship between attendance, follow-up weight, and individual-level characteristics. Most of the socio-demographic differences in weight loss are mediated by attendance to the programme. Examining further the predictors of attendance, the analysis revealed an inverted ‘U’ shape relationship between group size and attendance, with maximum attendance observed for groups of 15–18 people. Another important predictor identified was the number of days lapsed between referral and programme group start, indicating a decline of the ‘referral effect’ and the subsequent motivation to adhere to the programme. In contrast, neither scheduling (measured by time of the day and day of the week) nor programme familiarity of the instructor (measured by the number of past programme groups coached for each instructor) were associated with attendance.

This study adds to the existing literature on DPP by highlighting the importance of considering potentially modifiable influences on sociodemographic variation in response to such programmes. It demonstrates the importance of attendance as a potentially modifiable determinant of outcome. More research is needed to understand the modifiable drivers of attendance and retention, including specifically why groups who derive the least benefit from the DPP are more likely to attend fewer sessions. It points to the importance of efforts to maximise the implementation of existing DPPs to address health inequalities, in conjunction with efforts to create bespoke pathways for those groups that are much less engaged.
